# The Association of Gestational Age and Size with Management Strategies and Outcomes in Symptomatic Neonatal Tetralogy of Fallot

**DOI:** 10.1007/s00246-023-03365-w

**Published:** 2024-01-02

**Authors:** Leanne Duhaney, Martina A. Steurer, Rebecca Baer, Christina Chambers, Satish Rajagopal, Laura M. Mercer-Rosa, V. Mohan Reddy, Laura L. Jelliffe-Pawlowski, Shabnam Peyvandi

**Affiliations:** 1 Boston Children’s Hospital, Boston, MA USA; 2 University of California San Francisco, 550 16th Street, San Francisco, CA 94158 USA; 3 University of California San Diego, La Jolla, CA USA; 4 Children’s Hospital of Philadelphia, Philadelphia, PA USA

**Keywords:** Neonatal tetralogy of Fallot, Prematurity, Surgical outcomes

## Abstract

In neonatal, symptomatic tetralogy of Fallot (sTOF), data are lacking on whether high-risk groups would benefit from staged (SR) or complete repair (CR). We studied the association of gestational age (GA) at birth and z-score for birth weight (BWz), with management strategy and outcomes in sTOF. California population-based cohort study (2011–2017) of infants with sTOF (defined as catheter or surgical intervention prior to 44 weeks corrected GA) was performed, comparing management strategy and timing by GA and BWz categories. Multivariable models evaluated composite outcomes and days alive and out of hospital (DAOOH) in the first year of life. Among 345 patients (SR = 194; CR = 151), management strategy did not differ by GA or BWz with complete repair defined as prior to 44 weeks corrected gestational age; however, did differ by GA with regard to complete/timely repair (defined as complete repair within first 30 days of life). Full-term and early-term neonates underwent CR 20 (95%CI: − 27.1, − 14.1; *p *< 0.001) and 15 days (95%CI: − 22.1, − 8.2; *p *< 0.001) sooner than preterm neonates. Prematurity and major anomaly were associated with mortality or non-cardiac morbidity, while only major anomaly was associated with mortality or cardiac morbidity (OR = 3.5, 95%CI: 1.8,6.7, *p *< .0001). Full-term infants had greater DAOOH compared to preterm infants (35.2 days, 95%CI: 4.0, 66.5, *p* = 0.03). LGA infants and those with major anomaly had significantly lower DAOOH. In sTOF, patient specific risk factors such as prematurity and major anomaly were more associated with outcomes than management strategy.

**Supplementary Information:**

The online version contains supplementary material available at 10.1007/s00246-023-03365-w.

## Introduction

In a subset of neonates with symptomatic Tetralogy of Fallot (sTOF) (i.e. ductal dependent pulmonary blood flow, significant cyanosis, hypercyanotic spells), an intervention is necessary to provide a stable source of pulmonary blood flow. Early intervention can include complete, definitive repair or a staged/palliative approach through catheter or surgical techniques followed by later completion of repair. The literature to date suggests that there are benefits to both staged repair (SR) and complete repair (CR) in the neonatal period [1–5]. In particular, studies suggest that although there does not appear to be a difference in long-term mortality or significant morbidity based on initial management approach, SR may allow for better short-term survival [2]. Most studies have accounted for other patient-specific risk factors, such as prematurity in statistical models; however, a detailed examination of these risk factors influencing management and outcomes in this patient population is lacking.

The prevalence of congenital heart disease (CHD) increases with decreasing gestational age at birth [6, 7]. Thus, in the contemporary era, the field of congenital cardiology has seen a significant increase in premature and small infants presenting and being eligible for cardiac surgery due to increased postnatal survival rates [8]. However, this is a known high-risk population with excess mortality and morbidities noted in several studies [6, 9]. In a recent investigation using population-based data, we found that although mortality has decreased for premature infants with CHD requiring surgery, major neonatal morbidity (i.e. necrotizing enterocolitis and bronchopulmonary dysplasia) has increased [10]. Data are lacking regarding the optimal timing (neonatal vs. delayed) and optimal interventional approach (initial palliation vs. initial complete repair) among premature and small infants with CHD, including neonates with sTOF. Thus, there is significant practice variation across centers related to specific skills and general philosophies within an individual center.

Our primary aim for this study was to evaluate the association of gestational age at birth and birth weight, as measured by z-score for birth weight, and management strategies and outcomes among neonates with sTOF requiring an intervention.

## Methods

This was a retrospective population-based cohort study. We utilized the California Office of Statewide Health Planning and Development (OSHPD) database, now termed the Health Care and Access Information (HCAI) database. This database includes detailed information on infant characteristics derived from all California licensed hospital discharge records (birth hospitalization and readmissions) from birth to 1 year of life, as well as infant birth and death certificates from 2011 to 2017. The database includes linked information to maternal clinical and demographic characteristics. The file provides diagnosis and procedure codes based on the *International Classification of Diseases, Ninth Revision, Clinical Modification* (*ICD‐9‐CM*) and the *International Classification of Diseases, Tenth Revision, Clinical Modification (ICD-10-CM)*. The HCAI database has been used previously in multiple studies examining birth and outcomes in infants with CHD [6, 11–14]. Institutional review board approval was obtained from the Committee for the Protection of Human Subjects within the Health and Human Services Agency of the State of California, and the need for informed consent was waived.

We included all live born infants that met our definition of neonatal sTOF. The primary definition used to identify the study cohort included diagnostic codes consistent with TOF along with a procedure code for cardiac intervention that occurred prior to 44 weeks corrected gestational age during the neonatal hospitalization (Supplementary Methods includes all ICD-9 and ICD-10 codes considered for this classification scheme). To determine initial complete versus palliative repair, we used a similar algorithm as previously published by Savla et al. [15].

### Primary Exposure

The primary exposure was complete TOF repair before corrected gestational age of 44 weeks versus staged repair. To align with prior literature on this topic, we repeated the analyses using the more traditional definition requiring the complete repair to take place within the first 30 days of life (complete and timely repair) versus staged/delayed complete repair [1, 2].

Other primary exposure variables included z-score for birth weight (z-score for BW) categories and gestational age categories. The calculation of the z-score for BW category was completed using data published by Talge et al. [16] and described in previously published work by Steurer et al. [11, 17]. Z-scores were derived for birthweight by gestational age at birth and sex from U.S. live‐birth data provided by the National Center for Health Statistics (years 2009–2010). Appropriate for gestational age (AGA), small for gestational age (SGA), and large for gestational age (LGA) are commonly used categories when assessing fetal growth. They are defined as follows: small for GA (SGA; BW < 10th percentile for GA and sex, corresponding to a *Z* score <  − 1.27), adequate for GA (AGA; BW 10–90th percentile for GA and sex, *Z* score − 1.27 to 1.27), and large for GA (LGA; BW > 90th percentile for GA and sex, *Z* score >  + 1.27). Gestational age was categorized as: preterm (≤36 weeks), early-term (37–38 weeks), and full-term (>38 weeks). Another variable of interest was major anomaly, which refers to any major anomaly or major syndrome with the exception of cardiac defects.

### Outcome Variables

The primary outcomes included 1) a composite outcome defined as mortality *or* non-cardiac morbidity up to one year of age, and 2) a composite outcome defined as mortality *or* cardiac morbidity up to one year of age. Non-cardiac morbidity was defined as bronchopulmonary dysplasia (BPD), necrotizing enterocolitis (NEC), intraventricular hemorrhage (IVH) > grade II, periventricular leukomalacia (PVL), and retinopathy of prematurity (ROP) > stage 2. Cardiac morbidity was defined as mechanical support (ECMO), cardiac arrest, pacemaker, dialysis, diaphragm paralysis, tracheostomy or cerebral vascular event. As a secondary outcome, we also assessed days alive and out of hospital (DAOOH) in the first year of life.

### Statistical Analysis

Descriptive statistics were used to compare baseline demographic and other variables between the complete and staged repair groups. We then described surgical details including repair type and age at intervention by gestational age category (preterm, early-term and full-term) and by z-score for BW categories (AGA, SGA, LGA). Chi square was used to compare proportions and Kruskal–Wallis one-way analysis of variance was used to compare means. To assess the impact of gestational age at birth and z-score for BW on timing of neonatal intervention, we built multivariable linear regression models with Days of Life (DOL) at intervention as the outcome. These models included GA category and z-score for BW category. We stratified by repair type (any repair prior to 44 weeks corrected gestational age, complete repair prior to 44 weeks gestational age, and complete and timely repair prior to 30 days of age).

We built multivariable logistic regression models a priori to assess relationships between repair type (complete repair by 44 weeks corrected gestational age vs. staged repair) and binary outcomes (mortality, mortality or non-cardiac morbidity, and mortality or cardiac morbidity). Variables were chosen a priori for these models and included GA category, z-score for BW category and the presence of a non-cardiac major anomaly. For the continuous outcome, DAOOH, we used a multivariable linear regression model. We then repeated the multivariable models using the alternate definition of a complete TOF repair to be defined as a complete repair within the first 30 days of life regardless of corrected gestational age (i.e. complete and timely). With this alternate definition, our aim was to separate from this analysis infants for whom complete repair was completed after 30 days of life secondary to prematurity (i.e. infants who may have been on prostaglandin E1 (PGE1) during that time period).

Finally, we present Kaplan–Meier curves comparing the 1-year mortality of neonates who underwent complete timely repair versus delayed or staged repair. We stratified these analyses by GA and z-score for BW categories. We compared the curves with log rank tests.

All odds ratios (OR) were presented with 95% confidence intervals (CI), a p-value <0.05 was considered significant. All analyses were performed using STATA version 16.1 (Stata Statistical Software: Release 16. College Station, TX: StataCorp LP).

## Results

A total of 345 patients comprised the study cohort (complete repair = 151; staged repair = 94). Baseline demographics are listed in Table 1 by management strategy. Of the factors listed only median birthweight was different by management strategy (complete = 3090 g, IQR: 2525, 3460; staged = 2934 g, IQR: 2385, 3295, *p* = 0.03).

**Table 1:** Cohort demographics by surgical strategy

Factor	Categories	Staged repair (*n* = 194)	Complete repair (*n* = 151)	p-value*
Maternal age, median (IQR)		29.0 (24.0, 33.0)	30.0 (25.0, 33.0)	0.29
Maternal race/ethnicity, N (%)	Non-Hispanic White Hispanic Non-Hispanic Black Asian Other	58 (29.9%) 86 (44.3%) 8 (4.1%) 28 (14.4%) 14 (7.2%)	40 (26.5%) 68 (45.0%) 11 (7.3%) 17 (11.3%) 15 (9.9%)	0.51
Insurance, N (%)	Private Public	89 (48.9%) 93 (51.1%)	76 (52.1%) 70 (47.9%)	0.57
Gestational age (weeks), median (IQR)		38.0 (36.0, 39.0)	38.0 (37.0, 39.0)	0.30
Sex, N (%)	Male Female	114 (58.8%) 80 (41.2%)	74 (49.0%) 77 (51.0%)	0.071
Birth weight (grams), median (IQR)		2934.5 (2385.0, 3295.0)	3090.0 (2525.0, 3460.0)	0.033
BW Z-score categories, N (%)	AGA SGA LGA	143 (73.7%) 40 (20.6%) 11 (5.7%)	121 (80.1%) 23 (15.2%) 7 (4.6%)	0.37
Major extracardiac birth anomaly, N (%)	No Yes	168 (86.6%) 26 (13.4%)	123 (81.5%) 28 (18.5%)	0.19

We first evaluated the association of gestational age at birth and z-score for BW with the surgical management strategy. Table 2 demonstrates the association of GA category on surgical timing and management. Preterm infants had a lower corrected gestational age at the time of their first operation (38.14 weeks; IQR = 36.7, 39.9) compared to full-term infants (41.1 weeks; IQR = 40.1, 42, *p* = 0.0001), but DOL at the first operation was significantly later 26.5 days (IQR = 8, 53.3) compared to 10 days (IQR = 6, 18, *p* = 0.0001) in full-term infants. There was no statistically significant difference in management strategy across the GA groups, though the majority of preterm infants underwent a staged repair (62.5% vs. 37.5%, *p* = 0.29). When using the secondary definition of surgical management (timely/complete repair defined as complete TOF repair within the first 30 days of life vs. staged/delayed repair), we noted that premature infants were more likely to undergo a staged or delayed complete repair (i.e. >30 days of life) compared to a complete repair before 30 days of life (83.3% vs. 16.7%; *p *< 0.0001). Among z-score for BW categories, SGA infants were more likely to have their first intervention at a later DOL (18 days; IQR = 7,37) compared to AGA (11 days; IQR = 6, 25) or LGA (8 days; IQR = 7, 16) infants (*p* = 0.04). Surgical management approach using either definition was not different based on z-score for BW category (Table 3).

**Table 2:** Neonatal TOF Management by gestational age groups

	Gestational age group			
	Preterm (<36) *n* = 96	Early Term (37–38) *n* = 97	Full term (>38) *n* = 152	p-value
Corrected GA at first operation (weeks)	38.14 (36.7, 39.9)	39.4 (38.4, 41.1)	41.1 (40.1, 42)	0.0001
DOL at first intervention, Median (IQR) (days)	26.5 (8, 53.5)	13 (6, 24)	10 (6, 18)	0.0001
DOL at first surgery if first surgery complete, Median (IQR) (days)	39.5 (7, 60)	20 (7, 29)	13 (8, 19.5)	0.004
Surgical approach (1)				0.29
Staged n (%)	60 (62.5%)	50 (51.5%)	84 (55.3%)	
Complete n (%)	36 (37.5%)	47 (48.4%)	68 (44.7%)	
Surgical approach (2)				<0.0001
Staged/Delayed n (%)	80 (83.3%)	63 (64.9%)	88 (57.9%)	
Complete/Timely n (%)	16 (16.7%)	34 (35.1%)	64 (42.1%)	

*GA* gestational age, *DOL* day of life, *IQR* interquartile range

Surgical approach (1): Complete repair defined as complete repair prior to 44 weeks corrected gestational age

Surgical approach (2): Complete and timely repair defined as complete repair within first 30 days of life. Infants that had a complete repair as the first operation but after 30 days of life and before 44 weeks corrected GA were re-classified as staged/delayed repair

**Table 3:** Neonatal TOF Management by birth weight z-score category

	Birth weight Z-score category			
	AGA *n* = 264	SGA *n* = 63	LGA *n* = 18	p-value
Corrected GA at first operation (weeks)	40.3 (38.7, 41.5)	40.1 (38.8, 42.3)	40 (38.8, 41.6)	0.46
DOL at first intervention, median (IQR) (days)	11 (6, 25)	18 (7, 37)	8 (7, 16)	0.04
DOL at first intervention if complete repair, median (IQR) (days)	17 (8, 26)	19 (6, 35)	8 (6, 11)	0.11
Surgical approach (1)				0.37
Staged n (%)	143 (54.2%)	40 (63.5%)	11 (61.1%)	
Complete n (%)	121 (45.8%)	23 (36.5%)	7 (38.9%)	
Surgical approach (2)				0.21
Staged/Delayed n (%)	172 (65.2%)	48 (76.2%)	11 (61.1%)	
Complete/Timely n (%)	92 (34.8%)	15 (23.8%)	7 (38.9%)	

*AGA* appropriate for gestational age, *SGA* small for gestational age, *LGA* large for gestational age, *DOL* day of life, *IQR* interquartile range

Surgical approach (1): Complete repair defined as complete repair prior to 44 weeks corrected gestational age

Surgical approach (2): Complete and timely repair defined as complete repair within first 30 days of life. Infants that had a complete repair as the first operation but after 30 days of life and before 44 weeks corrected GA were re-classified as staged/delayed repair

In a multivariable model of both GA category and z-score for BW category, neither variable was associated with receipt of complete vs. staged repair. However, the odds of a complete and timely repair (defined as complete repair within the first 30 days of life) was significantly higher among early-term (OR = 2.58, 95%CI: 1.30, 5.12; *p* = 0.006) and term infants (OR =  = 3.50, 95%CI: 1.87, 6.57; *p *< 0.0001) compared to preterm neonates. DOL at first surgery was significantly earlier among early-term (beta =  − 16.8, 95%CI: − 21.6, − 12.1, *p *< 0.001) and full-term infants (beta =  − 20.1, 95%CI: − 24.4, − 15.8, *p *< 0.0001) compared to preterm infants. This pattern held true when assessing DOL at first operation if the first operation was a complete repair using either definition of complete repair (Supplementary Table 1). Surprisingly, z-score for BW did not affect surgical approach or timing.

We then evaluated the association of GA at birth and birth weight with the primary composite outcomes (mortality and cardiac/non-cardiac morbidity within the first year of life). We first assessed mortality as a single outcome measure. In the overall cohort, one-year mortality was 11.3% (39/345 patients). In a multivarible model of initial surgical strategy, z-score for BW category and GA at birth category, only the presence of a major anomaly was associated with an increased odds of 1-year mortality (OR = 2.75, 95%CI: 1.3,6.0, *p* = 0.01) (Supplementary Table 2). In contrast, when we assessed the primary outcome of mortality or a significant non-cardiac morbidity up to 1 year of life, we found that full-term infants were less likely to experience this outcome compared to preterm infants (OR = 0.48, 95%CI: 0.25, 0.93, *p* = 0.03). The presence of a non-cardiac major anomaly continued to portend a poor outcome. When we assessed the outcome of mortality or a significant cardiac morbidity only the presence of a non-cardiac anomaly was associated with an increased odds of the outcome (OR = 3.5, 95%CI: 1.8,6.7, *p *< 0.0001) (Table 4).

**Table 4:** Multivariable logistic regression models for composite outcome measure of (1) mortality *or* significant non-cardiac morbidity at 1 year and (2) mortality *or* significant cardiac morbidity at 1 year (models adjusted for all variables listed in table). Gestational age category and the presence of a major anomaly were associated with the outcome of mortality or significant non-cardiac morbidity at 1 year. In contrast, the presence of a major anomaly and being large for gestational age were associated with the outcome of mortality or significant cardiac morbidity at 1 year

	Composite outcome			
	Mortality or significant *non-cardiac* morbidity at 1 year (*n* = 345)		Mortality or significant *cardiac* morbidity at 1 year (*n* = 345)	
	OR (95% CI)	p-value	OR (95% CI)	p-value
Surgical approach				
Staged	Ref	Ref	Ref	Ref
Complete	0.69 (0.38,1.2)	0.21	0.78 (0.44,1.4)	0.38
GA Birth				
Preterm	Ref	Ref	Ref	Ref
Early term	0.55 (0.26,1.1)	0.11	1.21 (0.56,2.6)	0.63
Full term	0.48 (0.25,0.93)	0.03	1.3 (0.65,2.5)	0.47
Birthweight Z-score categories				
AGA	Ref	Ref	Ref	Ref
SGA	1.84 (0.95,3.58)	0.07	1.42 (0.71,2.83)	0.31
LGA	2.12 (0.70,6.4)	0.18	2.81 (0.99,7.9)	0.05
Major Extracardiac Anomaly				
Yes	2.17 (1.1, 4.3)	0.025	3.50 (1.8, 6.7)	<0.0001

Non-cardiac morbidity definition: bronchopulmonary dysplasia (BPD), necrotizing enterocolitis (NEC), intraventricular hemorrhage (IVH) > grade II, periventricular leukomalacia (PVL), and retinopathy of prematurity (ROP) > stage 2

Cardiac morbidity definition: mechanical support (ECMO), cardiac arrest, pacemaker, dialysis, diaphragm paralysis, tracheostomy or cerebral vascular event

*GA* gestational age, *AGA* appropriate for gestational age, *SGA* small for gestational age, *LGA* large for gestational age

In a multivariable analysis assessing our secondary outcome of DAAOH, we found that full-term infants had a higher number of DAOOH compared to preterm infants (beta = 35.2 days, 95%CI: 4.0, 66.5, *p* = 0.03). In addition, LGA infants compared to AGA infants and those with a non-cardiac major anomaly had significant lower DAOOH (LGA: beta =  − 60.6, 95%CI: − 112.1, − 9.0, *p* =  = 0.02; non-cardiac major anomaly: beta =  − 49.1, 95%CI: − 80.6, − 17.5, *p* = 0.002) (Table 5).

**Table 5:** Multivariable linear regression model assessing secondary outcome of days alive and out of hospital in first year of life. The model was adjusted for all variables listed in table. Prematurity, large for gestational age and those with a major extracardiac anomaly had significantly shorter days alive and out of hospital in the first year of life

	Days alive and out of hospital	
Surgical approach	Coeff (95% CI)	p-value
Staged	Ref	Ref
Complete	6.5 (− 16.8, 29.8)	0.59
Day of life at first surgery	− 0.12 (− 0.81, 0.57)	0.73
GA Birth		
Preterm	Ref	Ref
Early term	22.2 (− 10.8, 55.3)	0.19
Full term	35.2 (4.0, 66.5)	0.03
Birthweight Z-score categories		
AGA	Ref	Ref
SGA	− 16.14 (− 46.2, 13.9)	0.29
LGA	− 60.6 (− 112.1, − 9.0)	0.02
Major Extracardiac Anomaly		
Yes	− 49.1 (− 80.6, − 17.5)	0.002

*GA* gestational age, *AGA* appropriate for gestational age, *SGA* small for gestational age, *LGA* large for gestational age

**Fig. 1:**
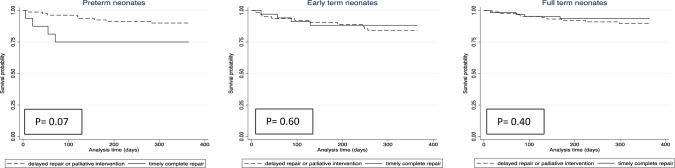
Kaplan Meier curves by surgical approach stratified by gestational age category at birth (preterm (≤36 weeks, *n* = 96); early term (37–38 weeks, *n* = 97); full-term (>38 weeks, *n* = 152). Definition used in this analysis: Timely and complete repair = complete repair within 30 days of life. Although not statistically significant, there appeared to be a visual difference suggesting lower survival probability in premature neonates that underwent a timely complete repair compared to those that underwent a delayed (i.e. >30 days of life) or palliative repair (*p* = 0.07)

**Fig. 2:**
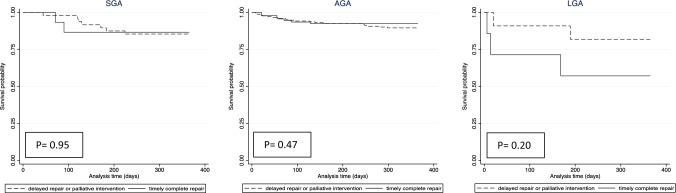
Kaplan Meier curves by surgical approach stratified by birth weight category (SGA = small for gestational age (*n* = 63); AGA = appropriate for gestational age (*n* = 264); LGA = large for gestational age (*n* = 18)). Definition used in this analysis: Timely and complete repair = complete repair within 30 days of life. Although not statistically significant, there appeared to be a visual difference on the Kaplan Meier curve suggesting lower survival probability up to 1 year of life in LGA infants that underwent a timely and complete repair compared to those that underwent a delayed (i.e. >30 days of life) or palliative intervention (*p* = 0.2)

We then repeated the analyses on outcomes using the secondary definition of complete repair (i.e. complete and timely repair defined as complete repair within first 30 days of life). There was no difference in primary and secondary outcomes compared to the original definition. We conducted a Kaplan–Meir analysis of survival probability up to 1 year of age stratified by GA birth category or z-score for BW category and stratified by delayed/staged repair versus timely complete repair (defined as complete TOF repair within the first 30 days of life). Among preterm infants, there appeared to be a visual difference on the Kaplan–Meier curves suggesting a higher survival probability for those that underwent delayed/staged management compared to those that underwent a complete and timely repair (defined as complete TOF repair within the first 30 days of life), however, there was not a statistically significant difference (*p* = 0.07) (Fig. 1). Additionally, although not statistically significant, LGA infants that underwent delayed/staged management also appeared to have a visual difference on the Kaplan–Meier curves suggesting a higher survival probability compared to those that underwent a timely and complete repair (defined as complete TOF repair within the first 30 days of life) (*p* = 0.2) (Fig. 2).

## Discussion

In this contemporary population-based study in California focused on neonates with symptomatic TOF, we identified the specific role of GA at birth and z-score for BW on management strategies and outcomes. In particular, we found that z-score for BW does not appear to influence management strategy, while prematurity (≤ 36 weeks) is associated with a lower likelihood of a complete and timely repair within the first 30 days of life. Despite this difference in management based on GA category, management strategy (complete vs. staged) was not associated with one-year outcomes. Instead, patient-specific risk factors, such as prematurity, LGA, and the presence of non-cardiac major anomalies were independently associated with one-year outcomes.

Our goals for this study were to first identify variations in practice patterns based on risk factors of prematurity and birth weight. In contrast to single center studies, the use of a population-based dataset of a large state like California allows for inclusion of several management strategies and variations in practice across centers when caring for this challenging patient population. Second, including infants that underwent any intervention prior to 44 weeks corrected gestational age (and not just within the first 30 days of life) allows for the inclusion of premature infants that may have been managed medically (i.e. prostaglandin) until an optimal age to undergo intervention. For example, a baby born at 30 weeks gestation may be medically managed with prostaglandin E1 (PGE1) for 6 weeks until a corrected gestational age of 36 weeks to allow for organ maturity and growth prior to intervention. If we had only used the traditional definition of neonatal intervention occurring within the first 30 days of life, this infant would have been excluded from our analysis. This approach allowed for the inclusion of many premature infants that would otherwise get excluded from similar analyses.

The observation that prematurity ≤ 36 weeks was associated with management strategy reflects either a preference for staged palliation (i.e. initial shunt or stent) or a longer duration of treatment with prostaglandin (>30 days after delivery) prior to undergoing a complete repair. This is not surprising given the technical challenges of cardiac surgery in premature infants [18, 19], the relative immaturity of other organ systems, and the known worse outcomes of premature infants with complex CHD [6, 17]. Previous reports have assessed prematurity as a binary variable and have shown similar findings related to management strategy [2]. Distinguishing between early-term and preterm infants is clinically important given previous studies demonstrating sub-optimal outcomes even in early-term infants [6, 20]. However, we did not find that early-term birth affected management strategy or outcomes in our study.

Taking into account these patient-specific risk factors, we did not find a difference in 1-year outcomes based on initial management strategy. This included the composite outcome of mortality or significant morbidity, and the number of days alive and out of the hospital. This finding is similar to previous reports from the Congenital Collaborative Research Collaborative (CCRC) in neonates with sTOF [2] and in a separate report among these neonates that were <2.5 kg [21]. However, we observed that other patient specific risk factors play a significant role in one-year outcomes. In particular, prematurity and the presence of another major anomaly are associated with increased risk of mortality or major non-cardiac morbidity, reflecting complications from prematurity in general. When assessing specifically the outcome of mortality or cardiac morbidity, prematurity was no longer associated with a worse outcome reflecting the unique morbidities that patients with prematurity face outside of cardiac complications (i.e. ROP, CLD) [6, 10]. We did not identify a statistically significant difference in outcomes based on management strategy in our multivariable logistic regression models, however, the stratified survival analysis seemed to demonstrate a visual difference suggesting higher survival probability among preterm infants that underwent a staged or delayed repair, though this was not statistically significant. Thus, in this particular group of high-risk patients a staged or delayed approach might be beneficial for longer-term outcomes; this should be studied in a larger database.

Interestingly, z-score for BW category was not associated with either definition used for complete repair. This might suggest that this clinical variable does not play a significant role in determining neonatal management within this study cohort. However, a novel finding in this study is the observation that LGA infants with sTOF appear to have worse outcomes (both composite outcomes as well as a lower number of days alive and out of hospital). LGA may reflect unique exposures in the maternal–fetal environment such as maternal diabetes that can influence not only somatic growth in utero, but also vulnerability to worse outcomes during high stress periods, such as cardiopulmonary bypass (CPB) [13, 22, 23]. In our survival analysis, LGA infants that underwent a staged repair (likely without the need for CPB in the neonatal period) seemed to demonstrate a visual difference suggesting higher survival probability compared to those that underwent a complete and timely repair; however, this finding was not statistically significant, likely as a result of a small sample size. Thus, sTOF neonates that are born LGA may benefit from a staged or delayed approach to avoid high stress events in the neonatal period leading to more optimal outcomes. This specific research question should be investigated further using national data registries.

The finding that SGA did not influence one-year outcomes is in contrast to previous reports identifying SGA as a significant risk factor for worse outcomes in infants with CHD [17] including a specific study on TOF neonates <2.5 kg that had a median follow-up of 5.5 years [21]. We elected to use z-score for BW as a measure of size at birth rather than the absolute measurement of birthweight. Birth weight and gestational age are collinear: a baby with low birth weight is most likely also of lower gestational age. Thus, z-score for BW assesses fetal growth independent of GA. It is well known in the neonatal literature and more recently from cardiac literature that z-score for BW has an important impact on outcomes of these neonates [17].

Our study has a few notable limitations. Given the use of administrative data, our study is prone to misclassification bias, though we would expect this bias to be non-differential. Second, although this is a population-based study we only include data from one state, which potentially limits the generalizability of our findings. Additionally, we are unable to include a center level variable in our analyses to adjust for practice variation across centers. We did not have reliable data in this database on the presence of 22q11.2 deletion syndrome, though we did include other major non-cardiac anomalies as a risk factor in our analyses. Finally, our analysis excludes neonates that may be discharged home from the hospital but then returned prior to 44 weeks corrected gestational age for intervention due to symptoms, though we expect this to be rare and not representative of the population. Despite these limitations, our study is strengthened by the use of contemporary (2011–2017) population-based data and the detailed categorization of gestational age at birth and birth weight. It is also important to highlight the possibility of unmeasured confounders in the study.

In conclusion, our study demonstrates that individual-level risk factors, such as prematurity, extracardiac anomalies, and LGA portend a higher risk for poor outcomes among neonates with symptomatic TOF, independent of management strategy. Although in the overall cohort, initial management strategy was not associated with outcomes, a staged or delayed approach in these high-risk groups may be considered to optimize long-term outcomes.

### Supplementary Information

Below is the link to the electronic supplementary material.

Supplementary file1 (DOCX 19 KB)

Supplementary file2 (DOCX 23 KB)
