# Supplementary material for: The Association of Gestational Age and Size with Management Strategies and Outcomes in Symptomatic Neonatal Tetralogy of Fallot
Source: Pediatr Cardiol. 2024 Jan 2;45(2):300–8. doi: 10.1007/s00246-023-03365-w (PMC10821998; doi:10.1007/s00246-023-03365-w)
Supplement: Supplementary file 1 — Supplementary file1 (DOCX 19 KB) [file 246_2023_3365_MOESM1_ESM.docx]

**Supplementary Table 1:** Multivariable linear regression on effect of gestational age at birth and birth weight z-score on timing of surgical repair as day of life of repair. Both gestational age category and birth weight z-score category were included in each model. 1) Any Operation: Day of life operation was significantly earlier for early term and term infants compared to preterm infants. Birth weight z-score category did not affect timing of surgery; 2) Complete repair only: restricting only to that that had a complete repair based on primary definition of < 44 weeks gestational age, day of life operation was significantly earlier for early term and term infants compared to preterm infants; 3) Complete and timely repair: restricting only to those that had a complete repair within the first 30 days of life (i.e. timely), day of life operation was significantly earlier for early term and term infants compared to preterm infants.

|  | **1) Any Operation (n= 345)** | |
| --- | --- | --- |
| **Variable** | **Coefficient** | **p-value** |
| GA birth |  |  |
| Preterm | Ref | Ref |
| Early Term | -16.8 (-21.6,-12.1) | <0.001 |
| Full Term | -20.1 (-24.4,-15.8) | <0.001 |
| Birth weight |  |  |
| AGA | Ref | Ref |
| SGA | 4.5 (-0.13,9.1) | 0.06 |
| LGA | -4.0 (-12.0,4.0) | 0.32 |
|  | **2) Complete Repair Only (n= 151)** | |
| GA Birth |  |  |
| Preterm | Ref | Ref |
| Early Term | -15.2 (-22.1,-8.2) | <0.001 |
| Full Term | -20.6 (-27.1,-14.1) | <0.001 |
| Birth Weight |  |  |
| AGA | Ref | Ref |
| SGA | 2.1 (-5.1,9.2) | 0.57 |
| LGA | -10.2 (-22.3,2.0) | 0.1 |
|  | **3) Complete *and* Timely Repair (n= 114)** | |
| GA Birth |  |  |
| Preterm | Ref | Ref |
| Early Term | 4.8 (-.50, 9.0) | 0.03 |
| Full Term | 4.6 (0.61, 8.52) | 0.02 |
| Birth Weight |  |  |
| AGA | Ref | Ref |
| SGA | -0.85 (-4.83,3.14) | 0.67 |
| LGA | -3.9 (-9.4,1.6) | 0.17 |

AGA= appropriate for gestational age; SGA= small for gestational age; LGA= large for gestational age; GA= gestational age

**Supplementary Table 2:** Multivariable logistic regression model for outcome measure of one-year mortality adjusting for all variables included in table. LGA infants had an increased odds of 1 year mortality compared to AGA infants. Infants with a major anomaly had an increased risk of mortality compared to those without an extracardiac anomaly.

|  | **One-year Mortality** | |
| --- | --- | --- |
|  | OR (95% CI) | p-value |
| Surgical Approach |  |  |
| Staged | Ref | Ref |
| Complete | 0.83 (0.41,1.70) | 0.61 |
| GA Birth |  |  |
| Preterm | Ref | Ref |
| Early Term | 1.4 (0.58,3.3) | 0.46 |
| Full Term | 0.7 (0.29,1.7) | 0.42 |
| Birthweight |  |  |
| AGA | Ref | Ref |
| SGA | 1.46 (0.63,3.4) | 0.38 |
| LGA | 3.64 (1.2,11.4) | 0.03 |
| Major Extracardiac Anomaly |  |  |
| Yes | 2.75 (1.3,6.0) | 0.01 |

GA= gestational age; AGA= appropriate for gestational age; SGA= small for gestational age; LGA= large for gestational age
