# Supplementary material for: The Association of Gestational Age and Size with Management Strategies and Outcomes in Symptomatic Neonatal Tetralogy of Fallot
Source: Pediatr Cardiol. 2024 Jan 2;45(2):300–8. doi: 10.1007/s00246-023-03365-w (PMC10821998; doi:10.1007/s00246-023-03365-w)
Supplement: Supplementary file 2 — Supplementary file2 (DOCX 23 KB) [file 246_2023_3365_MOESM2_ESM.docx]

1. Selection of all infants with tetralogy of Fallot (TOF) from the cohort with congenital heart disease

Infants were given a diagnosis of TOF if their record included the ICD-9 diagnostic code 745.2 or the ICD-10 diagnostic code Q21.3 irrespective of procedure codes. Infants without a diagnostic code for TOF who did not fit into any other heart lesion group were also assigned the diagnosis of TOF if they had ICD-9 or ICD-10 procedure codes for VSD closure *and* pulmonary valvulotomy/infundibulectomy (see codes below).

ICD-9

3513 "Open heart valvuloplasty of pulmonary valve"

3503 "Closed heart pulmonary valvotomy"

3534 "Infundibulectomy"

3553 "Repair of VSD"

3562 "Repair of VSD"

3572 "Repair of VSD"

ICD-10 (*means can have any ending)

027H04Z *or* 027H0DZ *or* 027H0ZZ *or* 02NH0ZZ *or* 02QH0ZZ or 13 “Dilation, repair or release of pulmonary valve”

02RM0*or 02UM0* or 02QM0* “VSD repair”

02BK0ZZ “Excision of right ventricle”

1. Restriction to TOF patients that underwent a neonatal procedure: We assessed procedure codes related to TOF before corrected 44 weeks of gestation. Any of the following codes qualified

| ICD-9 | Description | ICD-10 | Description | ICD-10 indicator |
| --- | --- | --- | --- | --- |
| 35.03 | Closed heart valvotomy, pulmonary valve | 02NH3zz | Release pulmonary valve, percutaneous approach | 38 |
| 35.13 | Open heart valvuloplasty of pulmonary valve without replacement | 027H04Z  *or*  027H0DZ  *or*  027H0ZZ  *or*  02NH0ZZ  *or*  02QH0ZZ  13 | Dilation of pulmonary valve with drug-eluting intraluminal device, open  *or*  Dilation of pulmonary valve with intraluminal device, Open  *or*  Dilation of pulmonary valve, Open  *or*  Release pulmonary valve, Open  *or*  Repair pulmonary valve, Open | 13 |
| 35.25 | Open and other replacement of pulmonary valve with tissue graft | 02RH07Z  *or*  02RH08Z  *or*  02RH0KZ  *or*  02RH47Z  *or*  02RH48Z  *or*  02RH4KZ | Replacement of pulmonary valve w/autologous tissue, open  *or*  Replacement of pulmonary valve w/zooplastic tissue, Open  *or*  Replacement of pulmonary valve w/nonautologous tissue, open  *or*  Replacement of pulmonary valve w/autologous tissue, percutaneous  *or*  Replacement of pulmonary valve w/zooplastic tissue, percutaneous  *or*  Replacement of pulmonary valve w/non-autologous tissue, percutaneous | 12 |
| 35.26 | Open and other replacement of pulmonary valve | 02RH0JZ  *or*  02RH4JZ | Replacement of pulmonary valve w/synthetic substitute, open  *or*  Replacement of pulmonary valve w/synthetic substitute, percutaneous | 12 |
| 35.53 | Repair of VSD with prosthesis | 02RM0JZ  *or*  02UM0JZ *or*  02UM3JZ  *or*  02UM4JZ | Replacement of ventricular septum w/synthetic substitute, open  *or*  Supplement ventricular septum w/synthetic substitute, Open  *or*  Supplement ventricular septum w/synthetic substitute, percutaneous  *or*  Supplement ventricular septum w/synthetic substitute, percutaneous | 7 |
| 35.62 | Repair of VSD with tissue graft | 02RM07Z  *or*  02RM0KZ  *or*  02RM47Z  *or*  02RM4KZ  *or*  02UM07Z  *or*  02UM0KZ  *or*  02UM38Z  *or*  02UM48Z | Replacement of ventricular septum w/autologous tissue, open  *or*  Replacement of ventricular septum w/nonautologous tissue, open  *or*  Replacement of ventricular septum w/autologous tissue, percutaneous  *or*  Replacement of ventricular septum w/nonautologous tissue, percutaneous  *or*  Supplement ventricular septum w/autologous tissue, open  *or*  Supplement ventricular septum w/nonautologous tissue, open  *or*  Supplement ventricular septum w/zooplastic tissue, percutaneous  *or*  Supplement ventricular septum w/zooplastic tissue, percutaneous endo | 7 |
| 35.72 | Other and unspecified repair of VSD | 02QM0ZZ  *or*  02QM3ZZ  *or*  02QM4ZZ | Repair ventricular septum, Open  *or*  Repair ventricular septum, percutaneous  *or*  Repair ventricular septum, percutaneous endo | 7 |
| 35.81 | Total repair of TOF | 02BK0ZZ  *with*  02NH0ZZ  *with*  02RM0JZ  *with*  02RP0JZ  *or*  02RQ0JZ  *or*  02RR0JZ | Excision of right ventricle, open  *with*  Release of pulmonary valve, open (any)  *with*  Replacement of ventricular septum with synthetic substitute, open (any)  *with*  Replacement of pulmonary trunk with synthetic substitute, open  *or*  Replacement of RPA with synthetic substitute, open  *or*  Replacement of LPA with synthetic substitute, open | 16  and  13  and  7  and  39 |
| 35.92 | Creation of conduit between RV and PA | 021K08P  Or  021K08Q  Or  021K08R  Or  021K09P  Or  021K09Q  Or  021K09R  Or  021K0AP  Or  021K0AQ  Or  021K0AR  Or  021K0JP  Or  021K0JQ  Or  021K0JR  Or  021K0Kp  Or  021K0KQ  Or  021K0KR  Or  021K0ZP  Or  021K0ZQ  Or  021K0ZR | Bypass RV to pulmonary trunk w/zooplastic tissue, open  Or  Bypass RV to RPA w/zooplastic tissue, open  Or  Bypass RV to LPA w/zooplastic tissue, open  Or  Bypass RV to pulmonary trunk w/autologous venous tissue, open  Or  Bypass RV to RPA w/autologous venous tissue, open  Or  Bypass RV to LPA w/autologous venous tissue, open  Or  Bypass RV to pulmonary trunk w/autologous arterial tissue, open  Or  Bypass RV to RPA w/autologous arterial tissue, open  Or  Bypass RV to LPA w/autologous arterial tissue, open  Or  Bypass RV to pulmonary trunk w/synthetic substitute, open  Or  Bypass RV to RPA w/synthetic substitute, open  Or  Bypass RV to LPA w/synthetic substitute, open  Or  Bypass RV to pulmonary trunk w/nonautologous tissue, open  Or  Bypass RV to RPA w/nonautologous tissue, open  Or  Bypass RV to LPA w/nonautologous tissue, open  Or  Bypass RV to pulmonary trunk, open  Or  Bypass RV to RPA, open  Or  Bypass RV to LPA, open | 11 |
| 35.96 | Percutaneous Valvuloplasty | 027H3ZZ  *or*  027J3ZZ | Dilation of pulmonary valve, percutaneous approach  *or*  Dilation of pulmonary valve, percutaneous endoscopic approach | 38 |
| 39.0 | Systemic to PA shunt | 021P08A  Or  021P08B  Or  021P08D  Or  021P0JA  Or  021P0JB  Or  021P0JD  Or  021P0KA  Or  021P0KB  Or  021P0KD  Or  021Q08A  Or  021Q08B  Or  021Q08D  Or  021Q0JA  Or  021Q0JB  Or  021Q0JD  Or  021Q0KA  Or  021Q0KB  Or  021Q0KD  Or  021R08A  Or  021R08B  Or  021R08D  Or  021R0JA  Or  021R0JB  Or  021R0JD  Or  021R0KA  Or  021R0KB  Or  021R0KD  Or  031309M  M and N indicate pulmonary artery  Or  031309N  Or  03130AM  Or  03130AN  Or  03130JM  Or  03130JN  Or  03130KM  Or  03130KN  03130ZM  Or  03130ZN  Or  031409M  Or  031409N  Or  03140AM  Or  03140AN  Or  03140JM  Or  03140JN  Or  03140KM  Or  03140KN  Or  03140ZM  Or  03140ZN | Bypass pulmonary trunk from innominate artery with zooplastic tissue, open  Or  Bypass pulmonary trunk from subclavian w/zooplastic tissue, open  Or  Bypass pulmonary trunk from carotid w/zooplastic tissue, open  Or  Bypass pulmonary trunk from innominate artery w/synthetic sub, open  Or  Bypass pulmonary trunk from subclavian w/synthetic sub, open  Or  Bypass pulmonary trunk from carotid w/synthetic sub, open  Or  Bypass pulmonary trunk from innominate artery w/ nonautologous tissue, open  Or  Bypass pulmonary trunk from subclavian w/ nonautologous tissue, open  Or  Bypass pulmonary trunk from carotid w/nonautologous tissue, open  Or  Bypass RPA from innominate artery w/zooplastic tissue, open  Or  Bypass RPA from subclavian w/zooplastic tissue, open  Or  Bypass RPA from carotid w/zooplastic tissue, open  Or  Bypass RPA from innominate artery w/synthetic sub, open  Or  Bypass RPA from subclavian w/synthetic sub  Or  Bypass RPA from carotid w/synthetic sub  Or  Bypass RPA from innominate artery w/nonautologous tissue, open  Or  Bypass RPA from subclavian w/nonautologous tissue, open  Or  Bypass RPA from carotid w/nonautologous tissue, open  Or  Bypass LPA from innominate artery w/zooplastic tissue, open  Or  Bypass LPA from subclavian w/zooplastic tissue, open  Or  Bypass LPA from carotid w/ zooplastic tissue, open  Or  Bypass LPA from innominate artery w/synthetic sub, open  Or  Bypass LPA from subclavian w/synthetic sub, open  Or  Bypass LPA from carotid w/synthetic sub, open  Or  Bypass LPA from innominate artery w/nonautloglous tissue, open  Or  Bypass LPA from subclavian w/nonautologous tissue  Or  Bypass LPA from carotid w/ nonautologous tissue  Or  Bypass R subclavian artery to RPA w/autologous venous tissue, open  Or  Bypass R subclavian artery to LPA w/autologous venous tissue, open  Or  Bypass R subclavian artery to RPA w/autologous arterial tissue, open  Or  Bypass R subclavian artery to LPA w/autologous arterial tissue, open  Or  Bypass R subclavian artery to RPA w/synthetic sub, open  Or  Bypass R subclavian artery to LPA w/synthetic sub tissue, open  Or  Bypass R subclavian artery to RPA w/non-autologous tissue, open  Or  Bypass R subclavian artery to LPA w/non-autologous tissue, open  Or  Bypass R subclavian artery to RPA, open  Or  Bypass R subclavian artery to LPA, open  Or  Bypass left subclavian artery to RPA w/autologous venous tissue, open  Or  Bypass left subclavian artery to LPA w/autologous venous tissue, open  Or  Bypass left subclavian artery to RPA w/autologous arterial tissue, open  Or  Bypass left subclavian artery to LPA w/autologous arterial tissue, open  Or  Bypass left subclavian artery to RPA w/synthetic sub, open  Or  Bypass left subclavian artery to LPA w/synthetic sub, open  Or  Bypass left subclavian artery to RPA w/non-autologous tissue, open  Or  Bypass left subclavian artery to LPA w/non-autologous tissue, open  Or  Bypass left subclavian artery to RPA, open  Or  Bypass left subclavian artery to LPA, open | 28 or 29 |
| 39.23 | Other intrathoracic vascular shunt or bypass |  | No appropriate conversions |  |
| 39.5 | Angioplasty or atherectomy of other noncoronary vessels |  | No conversion code |  |
|  | Ductal stent | 021X48P  Or 021X48Q  Or  021X48R  Or  021X49P  Or  021X49Q  Or  021X49R  Or  021X4AP  Or  021X4AQ  Or  021X4AR  Or  021X4JP  Or  021X4JQ  Or  021X4JR  Or  021X4KP  Or  021X4KQ  Or  021X4KR  Or  021X4ZP  Or  021X4ZQ  Or  021X4ZR | ***Following codes would denote a ductal stent:***  Bypass thoracic Ao, ascending/arch to pulm trunk w/zooplastic, percutaneous  Or  Bypass thoracic Ao, ascending/arch to RPA w/zooplastic, percutaneous  Bypass thoracic Ao, ascending/arch to LPA w/zooplastic, percutaneous  Or  Bypass thoracic Ao, ascending/arch to pulm trunk w/autologous venous, perc  Or  Bypass thoracic Ao, ascending/arch to RPA w/autologous venous, percutaneous  Or  Bypass thoracic Ao, ascending/arch to LPA w/autologous venous, percutaneous  Or  Bypass thoracic Ao, ascending/arch to pulm trunk w/autologous arterial, perc  Or  Bypass thoracic Ao, ascending/arch to RPA w/autologous arterial, perc  Or  Bypass thoracic Ao, ascending/arch to LPA w/autologous arterial, perc  Or  Bypass thoracic Ao, ascending/arch to pulm trunk w/synthetic sub, perc  Or  Bypass thoracic Ao, ascending/arch to RPA w/synthetic sub, perc  Or  Bypass thoracic Ao, ascending/arch to LPA w/synthetic sub, perc  Or  Bypass thoracic Ao, ascending/arch to pulm trunk w/nonautologous, perc  Or  Bypass thoracic Ao, ascending/arch to RPA w/nonautologous, perc  Or  Bypass thoracic Ao, ascending/arch to LPA w/nonautologous, perc  Or  Bypass thoracic Ao, ascending/arch to pulm trunk, percutaneous  Or  Bypass thoracic Ao, ascending/arch to RPA, percutaneous  Or  Bypass thoracic Ao, ascending/arch to LPA, percutaneous | 40 |

c) Defining complete repair: STATA codes used to identify infants that underwent complete neonatal repair based on various combinations of ICD-9 or ICD-10 procedure codes. This was based on the algorithm defined by Savla et al^15^.

//complete tof repair

generate full_repair = 1 if p_3581 ==1 //repair tof ICD9

replace full_repair = 1 if p_16 ==1 & p_13==1 & p_7==1 //ICD 10 equivalent to repair of tof

replace full_repair = 1 if p_16 ==1 & p_39==1 & p_7==1 //ICD 10 equivalent to repair of tof

//RVPA conduit and VSD closure

replace full_repair = 1 if p_3592 ==1 & (p_3553==1 | p_3562==1 | p_3572==1) // ICD9

replace full_repair = 1 if p_11 == 1 & p_7 == 1 //this is for icd10

//pulmonary valve repair/replacement and VSD closure

replace full_repair = 1 if (p_3513==1 | p_3525==1 |p_3526==1 | p_3534==1) & (p_3553==1 | p_3562==1 | p_3572==1) //ICD9

replace full_repair = 1 if (p_13==1 | p_12==1) & p_7 ==1 //this is for icd10
